# Entropy and its Application to Urban Systems

**DOI:** 10.3390/e21010056

**Published:** 2019-01-12

**Authors:** Ben Purvis, Yong Mao, Darren Robinson

**Affiliations:** 1 Laboratory for Urban Complexity and Sustainability, University of Nottingham, Nottingham NG7 2RD, UK; 2 School of Physics and Astronomy, University of Nottingham, Nottingham NG7 2RD, UK; 3 School of Architecture, University of Sheffield, Sheffield S10 2TN, UK

**Keywords:** entropy maximisation, second law, dissipative systems, cities, urban modeling, thermodynamics, information, statistical physics, probability

## Abstract

Since its conception over 150 years ago, entropy has enlightened and confused scholars and students alike, from its origins in physics and beyond. More recently, it has been considered within the urban context in a rather eclectic range of applications. The entropy maximization approach, as applied by Alan Wilson and others from the 1960s, contrasts with considerations from the 1990s of the city as a thermodynamic dissipative system, in the tradition of Ilya Prigogine. By reviewing the relevant mathematical theory, we draw the distinction among three interrelated definitions of entropy, the thermodynamic, the figurative, and the information statistical. The applications of these definitions to urban systems within the literature are explored, and the conflation of the thermodynamic and figurative interpretations are disentangled. We close this paper with an outlook on future uses of entropy in urban systems analysis.

## 1. Introduction

Oxford Dictionaries defines entropy in three categories: (1) physical: “a thermodynamic quantity representing the unavailability of a system’s thermal energy for conversion into mechanical work, often interpreted as the degree of disorder or randomness in the system”; (2) figurative: “lack of order or predictability, gradual decline into disorder”; (3) information statistical: “a logarithmic measure of the rate of transfer of information in a particular message or language” [1]. The first definition comes from physics, and it may be argued to be equivalent to a special case of the third, information statistical, definition, applied at the microscopic level [2,3,4,5]. It is the second definition that proves to be a source of much confusion since this figurative sense is often conflated with the more strictly defined physical and statistical senses. 

Entropy first entered the corpus of thermodynamics in the 1850s with Rudolf Clausius’s formulation of the Second Law of Thermodynamics, as a measure of the quality of heat energy in relation to temperature, and a characterization of irreversibility. Since then, the thermodynamic formulation has been shown to be equivalent to the molecular statistical formulations of entropy by Ludwig Boltzmann and Josiah Gibbs. In the 1940s, Claude Shannon introduced a statistical measure of “information content” which, due to its obvious similarities to the Boltzmann and Gibbs formulations, was also named entropy. The principle of maximum entropy, first formulated by E.T. Jaynes, states that, this statistical measure, when maximized subject to any known constraints, leads to the most likely distribution [2,6]. This statistical formulation, when applied at the microscopic level, is equivalent to the Boltzmann and Gibbs formulations. However, it can be applied more generally, especially in cases where details are either unknown or ill defined, such as in urban systems or cities.

Cities are complex and have often been viewed through the lens of systems in an attempt to better understand the numerous complex processes and structures that form a greater whole [7,8]. Significant attention is given to a plethora of interrelated urban issues such as segregation, pollution, and access to infrastructure and services, spanning all levels of complexity. The emergent non-equilibrium structure of the urban system depends on the interactions of its actors, whether the structure includes institutions, firms, households, or aggregations of these. The urban subsystems across which these actors interact may be classified in terms of the temporal scale over which they evolve, from slow, such as land use or transportation networks, to fast or immediate, such as employment or movement of people [9,10]. While there can exist local steady states, the system is dynamically evolving and we cannot predict an exact future state, but we may be able to produce models for likely configurations. With the prominence in recent years of ‘big data’ and increasing access to previously uncollected geographic data, new avenues exist for the calibration and testing of such urban models, as well as techniques to process and interpret this abundance of data [11]. It is in these areas that entropy has generated interest, in all its forms, be it as a statistical tool for analyzing these large urban datasets and performing inference where data is missing or incomplete, or purposed toward analyzing and understanding the irreversibility of complex urban processes. This latter focus is motivated by the recognition that cities dominate global resource use, with correspondingly adverse environmental impacts. The United Nations identifies “sustainable cities and communities” as one of its 17 Sustainable Development Goals [12].

The physical definition of entropy typically yields interpretation of the city as a thermodynamic system, identified within the literature particularly with regard to postulated links between entropy, the irreversibility of the second law of thermodynamics, and a notion of ‘sustainability’ [13,14,15,16,17,18]. This approach has its roots with both the work on non-equilibrium thermodynamics of Ilya Prigogine, and that of the economist Nicolas Georgescu-Roegen, who considered entropy in relation to the economic process [19,20]. It is often, however, presented qualitatively and can border on the second more figurative definition of entropy. The third, information statistical, definition has seen applied in a widespread manner to urban systems. For example, Alan Wilson’s employment of entropy maximization in the modeling of transport routing networks [21].

In this paper, we review how these interpretations of entropy have been applied to urban systems. In so doing we clarify and contextualize the differences among these three interpretations by outlining their scope of applicability. Section 2 presents the theoretical foundations of entropy in both its information statistical, and physical interpretations, highlighting the equivalence of the two. We present an overview of the theory in as concise a manner as possible, with an aim to cover the main bases for understanding the entropy concept. Section 3 reviews the applications of these statistical and thermodynamic interpretations respectively, in the context of urban systems. Lastly, in Section 4 and Section 5, we discuss the interrelations between the three entropy definitions, and offer an outlook on appropriate future uses of entropy in urban systems analysis.

## 2. Information and Entropy

Quantifying information content is relative, and context-specific, depending on the amount of information that is possessed by the observer. Shannon’s 1948 treatise proposes a measure of information content based upon the transmission of a message from one person to another [22]. In this scenario, the message, X, is conceived as a string of random variables, which can take on a number of states $x_{i}$, with a probability of occurrence given by $p_{i}$. Without observation, the receiver has no information, and perceives maximum uncertainty. The information provided by the observation of the state $x_{i}$ is a measure of how much uncertainty it resolves for the receiver. The lower the probability of $x_{i}$, the more information its observation provides. For example, if X is the sum of two unbiased dice rolls, the low probability observation X=2 implies 2 throws of 1, whereas X=7 is more ambiguous, and has a higher corresponding probability. In this sense, X=2 gives more information about the individual dice values than the high probability observation X= 7. We can call the most complete description of the system, which represents the results of each individual roll, a *microstate*. In contrast, the aggregate description of the sum of die values, X, represents a *macrostate* and may correspond to multiple microstates.

Axiomatically, Shannon showed that we can derive a measure for the information content of the form.

(1) $$H=\sum_{i}p_{i}\log\left(\frac{1}{p_{i}}\right)$$

where the sum is over all possible observations, and the base of the logarithm relays the units of information, e.g., base 2 gives rise to the unit of ‘bits’. This formulation follows intuitively from the discussion above, which is an observation that occurs with certainty, i.e., $p_{i}=1$, contains no information, $H\left(p_{i}\right)=\;0$. Additionally, the information obtained from two independent observations is additive while the joint probability is multiplicative: $H\left(p_{1}p_{2}\right)=H\left(p_{1}\right)+H\left(p_{2}\right)$. The logarithm, which is the only mathematical function that converts multiplication into addition, is the natural candidate for mapping probability p into information H. Shannon defines H as the entropy of the set of observation probabilities $p_{i}$. This quantity is described by Shannon as “a measure of how much ‘choice’ is involved in the selection of the event or of how uncertain we are of the outcome” [22]. 

It can be interpreted as the unpredictability of the observed state, or the expected amount of information obtained from a single observation.

### 2.1. Entropy Maximization

As presented by Cesario (1975), a rational gambler will bet on an outcome that has, to their knowledge, the highest probability of occurring [23]. For our dice example, this most probable outcome is the macrostate X=7, as it has the highest number of microstates, i.e., six different ways of occurring, assuming unbiased dice where each microstate has equal probability. This is equivalent to choosing the macro state with the maximum entropy. The principle of maximum entropy states that the probability distribution with the largest entropy, subject to known constraints, gives the best representation of our current state of knowledge of the system. To the gambler, this represents the probability distribution that is most likely to be ‘true’. If the gambler were to gain additional information, e.g., that one of the dice is biased, the perceived probability distribution and the most probable macro state could change. This new distribution may be determined by maximizing Equation (1) subject to any constraints given by this new information. If perfect information, i.e., a complete probability distribution providing the probabilities of each microstate, is given, the new most likely macro state would be easily determined. If this information is imperfect though, how does the gambler determine the best way to place a bet?

If no information about the probability distribution is known, the entropy function is maximized when all outcomes have the same probability, i.e., all $p_{i}$ are equal to 1/n. Otherwise, the gambler must use the probability distribution that maximizes the entropy subject to known constraints based upon all information that is available. This can be done formally by introducing Lagrangian multipliers, e.g., λ and μ for the two constraints of $\Sigma p_{i}=1$ and the expectation value of X, which leads to the general probability distribution $p_{i}=e^{-\lambda -\mu X}$. This form of probability can be used to determine the normalization constant, which leads to the partition function. This method can be generalized for any number of constraints, and is outlined in detail by Jaynes [2]. 

The principle of maximum entropy is profound, in that it uniquely determines the probability distribution that is maximally noncommittal to the missing information. It assumes no more than what is given, and provides a method for making inferences based upon the possession of partial information. The application of entropy maximization is understandably broad, ranging from statistical mechanics to image processing, genomic analysis, and any domain where data and information are assessed [24,25,26]. Jaynes concludes that statistical mechanics need not be regarded as a physical theory (such as classical thermodynamics), dependent for its validity on additional assumptions of mechanics or the principle of equal a priori probability (PEAPP), which stipulates that all accessible microstates are equivalent after all known constraints are considered. In other words, the method of statistical inference and maximum entropy is generally valid, regardless of the details of the system to which it is applied.

### 2.2. The Second Law and Thermodynamic Applications

In classical thermodynamics, Clausius states the second law of thermodynamics simply as “heat cannot spontaneously flow from a colder body to a hotter body”, which gives rise to the notion that the heat at higher temperatures is more useful and possesses a higher quality. He noted that, while the heat exchanged reversibly, $\int_{A}^{B}{\mathrm{dQ}}_{\mathrm{rev}}$, depends on the path taken from the initial state A to the final state B; division by the absolute temperature T produces an integral $\int_{A}^{B}\frac{{\mathrm{dQ}}_{\mathrm{rev}}}{T}$, which is independent of the path, and, therefore, corresponds to a function of state [27]. Clausius named this state variable entropy, S, defined through its differential, $dS=\frac{{\mathrm{dQ}}_{\mathrm{rev}}}{T}$, and $S_{B}-S_{A}=\int_{A}^{B}\frac{{\mathrm{dQ}}_{\mathrm{rev}}}{T}.$

The statistical mechanics formulation of entropy was hypothesized by Boltzmann through his famous equation $S=k_{B}\log W$, where $k_{B}$ is the Boltzmann constant and W is the number of microstates accessible to the system. The base of the logarithm is not critical and often taken to be e. The microstates are specific states of the system, which fixes all information (quantum or otherwise) for each individual atom or particle. For physical systems consisting typically of $10^{23}$ particles, W is of the order 2 to the power of $10^{23}$, which is an unimaginably large number. Hence, it is generally not possible to analyse each of those microstates, without the assumption that all accessible states are equivalent, PEAPP.

Boltzmann’s formula was later generalized by Gibbs for systems where microstates are occupied with probability $p_{i}$, with a sum over all available microstates.

(2) $$S=k_{B}\sum_{i}p_{i}\log\left(\frac{1}{p_{i}}\right)$$

For isolated systems, i.e., those with no means to exchange matter or energy with its surroundings, the assumption of PEAPP leads to $p_{i}=1/W$. Thus, the Boltzmann and Gibbs definitions of statistical entropy can be easily shown to be equivalent. For open systems (e.g., exchanging energy with the environment at temperature T), PEAPP gives way to the Boltzmann distribution, where a microstate with energy $E_{i}$ is occupied with probability $p_{i}$.

(3) $$p_{i}=\frac{1}{Z}{\;e}^{-E_{i}/k_{B}T},\;Z=\sum_{i}e^{-E_{i}/k_{B}T}$$

Suppose we have a reversible process where no work is exchanged and all microstates $E_{i}$ are preserved, the first law of thermodynamics dictates that the heat exchanged is the change in internal energy: ${\mathrm{dQ}}_{\mathrm{rev}}=\mathrm{dE}$, where $E=\sum_{i}p_{i}E_{i}$ is the total energy of the system. The Gibbs entropy definition can be differentiated to give (noting $\sum_{i}p_{i}=1$ and $\sum_{i}dp_{i}=0$):

(4) $$\mathrm{dS}=k_{B}\sum_{i}dp_{i}\log\left(\frac{1}{p_{i}}\right)$$

Combining with the Boltzmann distribution, we have (again noting $\sum_{i}dp_{i}=0$):

(5) $$dS=k_{B}\sum_{i}dp_{i}\left[\frac{E_{i}}{k_{B}T}-\log Z\right]=\sum_{i}dp_{i}\frac{E_{i}}{T}=\frac{\mathrm{dE}}{T}$$

Thus, the statistical entropy is equivalent to the classical definition due to Clausius. This equivalence is general, despite the proof relying on a reversible process without work exchange. This is due to the fact that entropy is a function of state and, therefore, does not have dependence on the specific process involved.

The Gibbs entropy is equivalent to Shannon’s definition, save for the Boltzmann constant, and classical thermodynamics may be viewed as an early example of the principle of entropy maximization. Ben-Naim argues that the units of ${\mathrm{JK}}^{-1}$ are but a “historical accident,” and that, if a new absolute temperature was defined as $\bar{T}=k_{B}T,$ the Gibbs and Shannon formulations would become equivalent [28] (pp. 204,205). Since the microstates are too numerous to assess individually, PEAPP represents the best interpretation of the physical system, and entropy is then maximized. Jaynes (1957) argues that we may view statistical mechanics as a special case of the more general procedures of inference derived from Shannon’s formulation of entropy [2]. In terms of information, we may, thus, think of Gibbs’ formulation as providing the amount of information needed to define the microstate of the system, given its macroscopic properties. Extensions of this equivalence in terms of quantum information and statistics may be found in References [4,5,29].

Given Boltzmann’s equation, $S=k_{B}\log W$, the maximization of entropy leads to the maximum number of microstates W, which correspond to the largest probability. Statistically, an isolated system will always evolve towards the most probable macro state, which corresponds to the largest number of microstates and maximum entropy. Thus, the second law can be interpreted statistically as, when heat flows from hot to cold, the number of accessible microstates increases such that the outcome “heat flows from hot to cold” is overwhelmingly more likely than “heat flows from cold to hot.” The second law is also equivalently stated as the “entropy of an isolated system always increases.” Jaynes formulates this in terms of information as “although our information as to the state of a system may be lost in a variety of ways, the only way in which it can be gained is by carrying out further measurements” [6].

It has been established from the 1920s by Eddington that the second law of thermodynamics holds a special position in science, in that it offers (at least for isolated systems) the arrow of time. No other law(s) in science explicitly distinguishes the past from the future. The second law is, therefore, often cited to imply a universal decline into disorder and chaos, so that entropy, itself an indicator of thermodynamic irreversibility, becomes naturally viewed as the indicator of this degradation. It should be noted that, in the thermodynamic context, the second law is firmly based on the statistics of large numbers of microstates (of the order of 2 to the power of $10^{23}$). This level of statistical significance is often lost in more general entropy applications. For example, we do not expect the second law to apply to social order, where possible states are both limited in number and open to interpretation.

## 3. Applications of Entropy to Urban Systems

Entropy, in all three definitions, has been applied to urban systems for a range of processes and phenomena. Without being exhaustive, we review and highlight some representative applications in this scenario. We split this discussion into applications of the information-statistical and physical thermodynamic interpretations of entropy, respectively, focusing particularly on the method of entropy maximization, and the characterization of a dissipative urban system as key examples of these two interpretations. The figurative definition is discussed in relation to conflation with the thermodynamic since it receives little application explicitly.

### 3.1. Information Statistical Entropy

There exist numerous applications of the information statistical definition of entropy to urban systems. A prominent family of these exploit the property that entropy is maximal when probabilities are evenly distributed, and zero when concentrated in a single location, and can, thus, be used for representing a measure of spatial concentration or dispersion [30]. Such an interpretation can yield numerous indices measuring such phenomena in urban systems as ethnic diversity [31], urban sprawl [32,33], segregation [34], diversity of urban land use [35], and the geographic distribution of species to infer biodiversity [36]. These applications are often coupled with GIS and remote sensing techniques to analyse real geographic data [37,38,39,40], as well as instance matching of similar points of interest through geo-location data [41]. Medvedkov too suggests a comparable entropy method in an attempt to find order in the spatial distribution of settlements by comparing random and clustered distributions [42]. An early review of such applications is presented in Reference [35], along with the discussion on when Shannon’s information measure that may be appropriately replaced with the alternate information measures of Brillouin and Good. These applications are often based on quite strong assumptions due largely to the difficulty in gathering the data needed to capture the distributions in the question.

Batty develops an information statistic termed ‘spatial entropy’ as a discretized formulation of Shannon’s continuous representation of information entropy, explicitly including the coordinate system through the use of the class interval [43,44,45]. The inclusion of the spatial interval size in Batty’s formulation holds implications for geographical analysis, and allows for a comparison of the effects of partitioning the spatial system, e.g., the explicit inclusion of zone size in entropy maximization models, and analysis of trends in the spread of probability distribution across an urban system.

These methods can of course also be applied to spatial scales beyond the urban, to facilitate the analysis of regional or national scales in e.g., ecology, or in economics to deal with quantities such as income inequality in a given population [46,47].

Wilson, through a series of works from the 1960s onwards, popularizes the application of entropy maximization to the urban region [21,48,49,50]. He casts the system of urban transport flows as, what Weaver (1948) refers to as, one of “disorganized complexity”. A system described by a large number of variables, with a large number of elements that interact only weakly [50,51]. In the context of modeling the pattern of transport flows within an urban system, the problem of finding “the most probable state” is initially posed. Dividing the system into zones between which travel occurs, a matrix $T_{ij}$ can be constructed, detailing all individual transport flows from zones i to j, describing the ‘state’ of the system. Wilson (1967) argues that a good estimate of $T_{ij}$ may be made by applying three constraints: fixing the total number of workers living in a given origin zone i, fixing the total number of jobs in a given destination zone j, and fixing the total ‘generalised cost’, or impedance, associated with travel to work [48]. By assuming that each microstate of $T_{ij}$ is equally probable, we, therefore, want to find the $T_{ij}$ with the largest number of microstates $W\left(T_{ij}\right)$ giving rise to it. This may be achieved by maximising $W\left(T_{ij}\right)$ subject to the three imposed constraints, although Wilson chooses to equivalently maximize logW, to allow for Stirling’s approximation to simplify the maths.

This function is then maximized subject to the given constraints using a Lagrangian multiplier approach, which reveals an exponential expression for the most probable $T_{ij}$ matrix. The resultant expression reduces to the function that had been previously utilised in earlier ‘gravity’ models, providing an independent theoretical derivation, and superseding these expressions without the need for arbitrary tuning constants [52,53]. The approach builds on the entropy maximization method developed by Jaynes and detailed above, down to the analogous search for the most likely macrostate, as emphasized by Wilson in References [21,49] and Reference [54]. He interprets the entropy, logW, as a measure of the system uncertainty, which ought to be maximized to give the most likely scenario. All knowledge of the system is considered as constraints of the maximization and are incorporated via the Lagrangian multipliers.

This derivation led Wilson to propose a family of ‘spatial interaction’ models based upon this method, but applying different constraints. These include a retail model where the destination is fixed and the flow to this constrained location becomes the subject of analysis, and various considerations of disaggregation of the trips among various archetypal groups [21,55]. This entropy maximization approach to urban transport flows has been picked up, expanded, and adapted by numerous authors over the years. Such considerations include a continuous rather than discrete spatial representation [56], and simultaneous minimization of the generalized cost function in order to optimize network topology [57]. The incorporation of prior information from external data, such as traffic counts, has been demonstrated by References [58,59,60]. In this case, not all trips are assumed equally likely since this ‘known’ information about the $T_{ij}$ matrix is built in as a constraint. Such methods provide ways of improving model accuracy based upon real world data. Griffith & Jones (1980) investigate the relation of distance decay to the spatial structure associated with the origins and destinations [61], and Mattson describes an approach for maximizing ‘welfare’ in the allocation of housing [62]. More comprehensive reviews of various adaptions and applications of this model are presented in References [57,63] as well as in Wilson’s 2010 reflections on the technique, where it is claimed that such methods are routinely used by international companies wishing to optimize the location of new retail site locations [50].

### 3.2. Thermodynamic Entropy

Applications of thermodynamic principles to the urban system are diverse. A review of some of these is presented by Filchakova et al [14]. For example, in engineering thermodynamics, exergy is defined as the maximum amount of work that may be obtained from a system by bringing it into equilibrium with its environment [64]. Historically, exergy analysis has been used to improve the thermodynamic efficiency of various industrial processes, by identifying and minimizing exergy destruction. This has led to applications to larger systems assessing energy efficiency at national, regional, and urban scales [65,66,67,68,69]. For example, Nielsen and Jørgensen (2015) develop an exergy accounting framework, mapping the locations of large exergy consumption across six societal sectors for a small region, which allowed for the identification of key areas of attention for a proposed ‘sustainable energy’ transition plan [68]. It can be shown that the exergy destruction of a process, $\Psi_{D}$, is related to its entropy production, $S_{G}$, through the environmental reference temperature, $T_{0}$, $\Psi_{D}=T_{0}S_{G}$ [70]. Thus, similar approaches considering entropy as a geographically applied indicator for targeting and improving upon energy inefficiencies are presented in References [71,72,73]. These methods remain superior to traditional energy accounting methods, as they capture the ‘quality’ of energy, that is its capacity to perform work [74,75]. 

One approach to modern thermodynamics, which departs somewhat from the familiar statistical mechanics interpretation, is an extension of classical thermodynamics outside of equilibrium, and has received attention in its purported applicability to urban systems. The vast majority of systems observed in nature are open, out of equilibrium, and undergoing irreversible processes. The urban system itself is one such example. Classical thermodynamics, concerned only with the initial and final states of systems in thermodynamic equilibrium, fails to include a theory of irreversible processes. A thermodynamic study of real systems requires a more general approach, something developed from the beginnings of the 20th century by theorists such as Onsager and Prigogine. However, this area of thermodynamics is still an active work in progress and lacks an established corpus [27]. Prigogine, in his study of the phenomena of self-organization, forwards the notion of ‘dissipative systems’ to refer to complex open structures that maintain their functioning through the constant dissipation of thermodynamic entropy [20]. This usually involves consideration of the entropy balance representing the total entropy change of the system as the respective sum of its internal entropy production and entropy exchange due to fluxes of matter and energy across the system’s boundary:

(6) $$\frac{dS}{dt}=\frac{d_{i}S}{dt}+\frac{d_{e}S}{dt}\;.$$

The second law tells us that the internal entropy production $d_{i}S/dt>\;0$, but the total entropy change of the system is permitted to be positive or negative, as $d_{e}S/dt={\;d}_{e}S\left(\mathrm{in}\right)/dt-d_{e}S\left(\mathrm{out}\right)/dt$ may take on either sign.

Consideration of the city as a dissipative system in relation to a notion of sustainability is considered by Rees & Wackernagel (1997) [13]. They describe cities as “entropic black holes”, which draw in large amounts of entropy and matter, and “export the resultant entropy (waste and disorder)” to maintain their “highly-ordered dissipative structure.” It is argued that the ordered dissipative structure of the city is maintained at the expense of “increasing entropy or disorder in the environment”, citing trends of natural capital depletion, pollution emissions, and other adverse anthropogenic ecological consequences. Marchettini et al. (2006) extend this thread of work, but caution against an “entropic euthanasia” where the urban system has fully degraded all potentials through unprecedented growth in energy and matter inflows, leading to “maximum disorder and maximum entropy” analogous to an ‘urban heat death’ [76]. Similar arguments are also explored in Reference [77].

Filchakova et al. (2007) consider similar arguments in their wider review of thermodynamic concepts applied to the city, exploring its casting as an open system, and the analogy of the city as a living organism [14]. Following the arguments put forward by Marchettini et al., they appear to strive toward a more quantitative thermodynamic representation to “represent urban metabolism in an operational way”. A suggestion of coupling ecosystem theory literature with that on urban metabolism is suggested, but no concrete steps are taken. 

Fistola (2011,2012) draws on authors such as Lovelock [78] and Rifkin [79], who both link entropy and the second law to notions of sustainability and humanity’s impact on global resources and ecosystems. A qualitative description of excessive levels of pollution is presented as a manifestation of “high entropy” and a loss of social capital due to increasing individualization, which is linked to an entropic trend of “structural decay” [80,81]. A strategy is articulated, which includes the assessment of “urban entropy”. This is developed in Reference [18] where a list of “entropy indicators” is produced including air quality, unemployment rate, waste production, and flooding risk, which are then mapped spatially. A similar approach using ‘entropy indicators’ is also taken by Pelorosso et al. in Reference [82]. These studies, while touching on the thermodynamic definition of entropy, employ the second, figurative, definition of entropy as a broader metaphorical gauge for a lack of ‘order’. 

There are two broad arguments here, the first being that to maintain ‘order’, dS/dt≤0, the urban system must export entropy to its surroundings, which, in turn, acts to degrade this external environment. The second, not necessarily incompatible with the first, argues that, in some cases, the entropy of the urban system itself grows, dS/dt>0, which threatens its functional integrity. Potentially, both arguments are based upon mixed premises regarding conflation of the thermodynamic and figurative definitions through the association of irreversibility with ‘degradation’. Despite Filchakova et al.’s call for more quantitative conceptualizations of these representations, this remains largely absent. Where indicators have been presented, their link to ‘entropy’ remains qualitative, containing none of the thermodynamic theory either outlined above or in more classical considerations despite continued interest within the literature. Why is this still the case?

We argue that this derives primarily from conflation between the first two definitions of entropy detailed in our introduction, the thermodynamic sense, and the figurative sense. This can be seen in the language that tends to be used, centered on a notion of ‘disorder’ and ‘degradation’, beyond a simple thermodynamic context. As has been pointed out by authors in the past, anything described as a source of ‘disorder’ such as pollution cannot be simply coded as ‘entropic’ or acting to increase entropy in the thermodynamic sense [73,83,84]. Entropy is not a direct measure of ‘utility’ or the lack of. For example, the increase in entropy due to the release of an amount of hydrogen cyanide into the environment is roughly equivalent to the same amount of carbon dioxide released, despite the potentially catastrophic effects of the former. Thus entropy, at least in its thermodynamic sense, is not a sufficient measure of the negative externalities caused by human activity, and the association of the sign of dS/dt to these is problematic. 

Furthermore, as has been pointed out by numerous authors, in conflating definitions 1 and 2, it is common to overlook the fact that the second law, cast in terms of inevitable decay to equilibrium or ‘heat death’, only applies to isolated systems that cannot exchange matter or energy with their environment [73,84,85]. Both the city, and Earth itself, are open systems, and thus a fear of ‘entropic accumulation,’ in the physical sense, is unfounded, since entropy produced is merely radiated out of the system as waste heat. As shown by e.g., Weiss (1994), the natural entropy production of the Earth system through the dissipation of solar energy greatly dwarfs any entropy produced by anthropic processes [86]. Of course, on an urban scale, this anthropic entropy production is arguably less negligible. The problems caused by urban heat islands are well documented, but these are much better understood in terms of other thermodynamic concepts such as heat, and we must understand the limits of entropy conceptualization where other such concepts take over. 

## 4. Discussion

In reviewing various applications of ‘entropy’ to urban systems, we have shown that the distinction between the three definitions laid out in the Oxford Dictionaries is not so clear within the literature. This can be seen primarily in conflation between the physical definition and the figurative one. This is something that has arguably been persistent since these definitions were first popularised, and the association of thermodynamic entropy with disorder was first put forward by Boltzmann and Helmholtz in the 19th century [87]. The analogy is often put forth with examples of a deck of cards becoming less ordered when shuffled, or a child’s room becoming messier over time. This offers an intuitive understanding of the abstract quantity of entropy that is appealing, but also demonstrably misleading. A full review of the merits and problems of the disorder metaphor is presented in Reference [88] Overreliance on this metaphor leads to qualitative perceptions of ‘disorder’ being presented as evidence of entropy, as seen in the discussions on dissipative systems above. This figurative use is widespread, but becomes problematic when one attempts to ‘operationalize’ it. It is for this reason perhaps that actual implementations of measuring thermodynamic entropy in this way, as noted by Filchakova et al., appear to be almost absent from the literature.

It seems that many authors are not aware of the clear distinctions between the first and second definitions. This is particularly apparent in the writings, and subsequent criticisms, of the economist Georgescu-Roegen, whose initial work relating entropy and the economic process underwent significant revisions over the course of his career [89,90,91]. He later reflects on how, delving into the field as an interdisciplinary pioneer, he was inspired by the writings of Max Planck on entropy and “matter dissipation”, leading to his proposed ‘fourth law’, the postulate that complete recycling of matter is impossible [92]. This body of work was heavily criticized by authors from the natural and social sciences alike, and its status is now widely regarded as overreaching in its application of thermodynamics, in part due to the inapplicability of thermodynamic entropy to material flows [91,93]. Despite this, Georgescu-Roegen’s challenge to the economic orthodoxy from an environmental standpoint is seen as a defining moment in terms of the founding of the field of ecological economics [94,95,96]. This presents a good example of wider confusion that pervades applications of thermodynamic entropy, and how the distinction between its figurative use as an analogy, and proposed operationalization as a well-defined physical quantity, is not always clear.

Clear parallels can be seen here regarding the work conceptualizing urban dissipative systems, particularly with regard to seeking wider applications of concepts traditionally limited to physics. These conceptualizations tend to rely on the analogy of the urban system to an organism or complex ecosystem, which itself predates considerations of thermodynamics in this respect, e.g., discussions of urban metabolism in Reference [97]. In a similar vein, reflections of biological systems in relation to entropy and the second law have received notable attention including Schrödinger’s 1944 ‘What is Life?’ [98], as well as considerations of entropy throughput as an indicator of ecosystem integrity or adaptive capacity [99,100,101]. These links provide the motivation to explore the possibilities of extending similar analyses to urban systems, particularly in relation to issues of sustainability and resilience. It seems however that, in translation across disciplines and the ambiguity of application vs. analogy, it becomes common to misplace much of the thermodynamic theory in favor of more figurative understandings of entropy. 

This holds some implications for any application of the first thermodynamic definition of entropy to urban systems. First, one should be careful that conflation is not being made with the second figurative definition. A general rule of thumb is that the first definition is measurable, at least in theory, whereas the figurative definition is not. To be of any use as an indicator one must be able to measure thermodynamic entropy in thermodynamic units of ${\mathrm{JK}}^{-1}$. Without a mathematical formulation, it remains a metaphor, and one which we argue fails to be useful, sowing confusion rather than bringing clarity. 

On the other hand, some authors present explicitly analogous mathematical constructs to thermodynamic and statistical mechanical principles to bring insights into other fields, including within ecology and urban analysis [101,102,103]. This has the potential to provide novel modeling techniques and insights, as the urban gravity-analogous models did in the mid-20th century. Caution should be taken however, regarding the limits to which such analogies can apply. In fundamental thermodynamics concepts like energy and the second law are well defined, where microscopic fluctuations are suppressed by the statistics of very large numbers. This is often not the case when seeking analogous thermodynamic ‘laws’ that govern social and ecological interactions, where fluctuations can determine overall system behaviour at this macroscopic scale.

What does this all mean for applications of the third information statistic definition? Generally this sense seems to remain distinct from the other two in the literature, although a small number of applications exist that attempt to combine entropy maximization with the language of dissipative systems [104,105]. As shown above, this third definition remains well-established in the literature considering urban system models, both as a measure for geographic dispersion, and for its use in entropy maximization techniques. We have argued that the first definition can be interpreted as a special case of the third definition, and is widely regarded in the literature. Despite this, these applications remain largely separate in consideration at the urban level. This can be largely seen as a matter of scale. The thermodynamic interpretation is concerned with specific microstates, the occupation of which are set by the Boltzmann distribution via energy levels of the microstates. PEAPP is often implicitly assumed. In this context, the statistics are drawn from the incredibly large number of microstates (of the order of 2 to the power of $10^{23}$) so that energy and temperature play central roles. In contrast, information statistical applications generally deal with macroscopic configurations (such as transport routing), whose combinations are significantly fewer in numbers. With systems often out of equilibrium, the equivalents of energy and temperature are less clear.

Conflation of the third statistical definition with the second figurative definition are less prevalent and the analogy with the disorder is less relevant [106]. As well as being a misleading and subjective metaphor, the concept of disorder lacks the axiomatic properties such as additivity from which Shannon derived his expression for H. Nevertheless, Ayeni (1976) cautions an analogous confusion of the technical meaning of ‘information’ with its everyday understanding [7]. One should note that information entropy, in its standard form, lacks any notion of ‘information quality’, cf. the discussion above of thermodynamic entropy was blind to notions of ‘utility’. Although interpretation here is often influenced by the choice of variables, this has implications for when population similarity cannot be assumed, where methods incorporating population weighting must be employed [107]. As emphasized by e.g., Walsh and Webber (1977), one must remember that entropy as a measure of information is not an objective measure of the system, but is dependent on the observer’s ‘model’ of the system and the experiments they choose to perform on it. In this case, one must be clear in the definition of entropy in terms of the system variables chosen, and avoid conflation between prior information about the system before experimentation and information obtained from an experiment [35]. 

While, in some circles, the entropy maximization approach has been fully embraced, others remain critical, and it may still be argued to form a fringe within human geography [50]. Central to much of this scepticism is the underlying epistemological assumptions. Not only that human actors at some level of statistical aggregation may be considered to operate rationally and deterministically like molecules, but also that a complex social system such as a city may be adequately described by a finite series of quantitative parameters [23,108,109]. This philosophical debate is beyond our scope, and so long as all modeling assumptions are laid out bare, we feel, largely moot.

## 5. Conclusions

The three notions of entropy have given rise to much general confusion, which overshadow its applications in urban systems. Disentangling these separate notions enables the concept of entropy to be utilised in a wide range of worthwhile applications. The first thermodynamic definition of entropy applies to specific microstates, and the assumption of PEAPP enables it to be connected to physical parameters such as energy and exergy. In this sense, entropy could supplement and enhance traditional energy/exergy analysis such as in diagnosing energy efficiency within the urban system [73]. It could also offer analogous use of thermodynamic identities for modeling urban phenomena. The second definition of entropy, as figurative only, should not feature in quantitative analysis, lest the first definition slide into this more metaphorical interpretation of entropy. The third, information statistical definition of entropy is the most general of the three, it yields numerous applications to urban systems, ranging from spatial clustering analysis of various phenomena, to performing statistical inference for incomplete datasets. At the microscopic scale of atoms, the statistical entropy coincides with the thermodynamic entropy, and the statistics of large numbers leads to the classical laws of thermodynamics. As a universal measure, information statistical entropy is applicable at all scales, and therefore it should perhaps be featured more extensively in urban studies than it does currently. In part, this may be due to the confusion surrounding its varying usages. By disentangling these definitions, we believe entropy in this context offers a huge potential for urban systems analysis in the future.
